# Neuromuscular Electrical Stimulation Plus Nutritional Counseling Attenuates Thigh Muscle Thickness Loss in Hospitalized Cancer Patients

**DOI:** 10.3390/pathophysiology32040068

**Published:** 2025-12-02

**Authors:** Tatyanne L. N. Gomes, Thaís C. Borges, Jessica F. M. Ivo, Lara G. Mainardi, Renata G. C. Abadio, Benjamin T. Wall, Gustavo D. Pimentel

**Affiliations:** 1 Faculty of Nutrition, Federal University of Goiás, Goiânia 74605-080, Brazil; thaisborges2041@gmail.com (T.C.B.); jfmivo@gmail.com (J.F.M.I.); gmainardilara@gmail.com (L.G.M.);; 2 Department of Sport and Health Sciences, College of Life and Environmental Sciences, University of Exeter, Exeter EX4 4PS, UK

**Keywords:** cancer, nutritional counseling, muscle mass, hospitalization, electric stimulation

## Abstract

Background and aims: This study aimed to determine whether neuromuscular electrical stimulation (NMES) combined with nutritional counseling promotes an increase in thigh muscle thickness (MT), as well as to assess changes in the relationship between MT and intracellular water (ICW). Body composition methods such as ultrasound may overestimate muscle mass, depending on the context, because they cannot distinguish the contractile protein component from body fluids, including intra- and extracellular water. Methods: A pilot randomized parallel trial was conducted with 25 hospitalized patients with unselected cancer, who were divided into two groups: NMES + Diet and Diet. Both groups received nutritional counseling, but only one group received NMES. NMES was applied bilaterally to the origin and insertion points of the quadriceps twice daily, with a 3 h interval between sessions, for 7 consecutive days. MT and ICW were measured before and after the intervention. Food consumption was assessed using a 24 h dietary recall at baseline and at the end of the study to quantify and adjust macronutrient intake during the intervention. Results: Both treatment groups (Diet × NMES + Diet) showed similar dropout rates which means participants in the more intensive treatment did not quit more frequently, once intervention with NMES was feasible and well tolerated. In addition, both groups showed a reduction in carbohydrate intake (*p* = 0.012) and an increase in leucine intake (*p* < 0.001) post-intervention. The increase in leucine intake was significantly greater in the NMES + Diet group (*p* < 0.001), and the reduction in carbohydrate intake was also greater in this group (*p* = 0.012). In the delta analysis, the NMES + Diet group showed an increase in thigh MT, whereas the Diet group experienced a decrease (Diet group: ∆ = −2.53 ± 3.73 mm vs. NMES + Diet group: ∆ = 2.09 ± 2.27 mm, *p* = 0.001). Moreover, the MT/ICW ratio was higher in the NMES + Diet group post-intervention (Diet group: ∆ = −0.15 ± 0.19 mm/L vs. NMES + Diet group: ∆ = 0.11 ± 0.09 mm/L, *p* < 0.001), while no significant difference in ICW was observed between groups. Conclusions: short-term intervention combining nutritional counseling with NMES increased thigh MT and the MT/ICW ratio, possibly due to NMES-induced extracellular water expansion.

## 1. Introduction

In patients with cancer, the presence of a tumor induces systemic metabolic reprogramming that can progress to cachexia [1]. The release of inflammatory and catabolic mediators by the tumor leads to anabolic resistance to protein synthesis and increases muscle catabolism [2]. Consequently, the loss of muscle mass results in intracellular dehydration [3,4] and protein catabolism [5].

Furthermore, muscle mass reduction is associated with several complications, such as prolonged hospitalization, adverse reactions to cancer treatment, an increased incidence of infections, reduced quality of life, and higher mortality [6]. Patients with cancer experience accelerated muscle mass loss, which may be exacerbated by treatment and disease progression [7]. Thus, nutritional support and physical exercise are considered fundamental strategies to maintain muscle mass, as they exert synergistic anabolic effects that help prevent or reduce muscle wasting [8].

Dietary protein intake can stimulate muscle protein synthesis even in the presence of anabolic resistance in patients with cancer; however, a higher protein intake is required compared with healthy individuals [9,10]. High-quality dietary proteins, such as those rich in leucine, may also provide greater stimulation of muscle protein synthesis [10]. The European Society for Clinical Nutrition and Metabolism (ESPEN) guidelines recommend that patients with cancer consume 25–35 kcal/kg and at least 1 g, ideally up to 1.5 g, of protein per kg of body weight per day to minimize muscle and weight loss and to effectively combat malnutrition [9].

In the absence of physical activity, the progressive loss of muscle mass due to disuse, in addition to the pathophysiological effects of the disease, can negatively affect motor neuron function, which is essential for muscle performance [11]. In this context, neuromuscular electrical stimulation (NMES) has emerged as a promising strategy for patients who cannot perform conventional physical exercise, as it induces muscle contraction through electrical impulses [7]. A single session of NMES has been shown to stimulate muscle protein synthesis [12] and attenuate muscle loss in healthy adults subjected to limb immobilization [13].

Although NMES has been successfully used in patients with cancer to alleviate muscle loss and improve strength [14,15,16,17,18], no study has evaluated the potential benefit of NMES combined with nutritional counseling on the ratio of muscle thickness to intracellular water volume (MT/ICW) in hospitalized patients with unselected cancer. Body composition assessment methods such as ultrasound may overestimate muscle mass because they cannot differentiate the contractile protein component from body fluids, including intra- and extracellular water [19]. Therefore, the aim of this study was to determine whether NMES combined with nutritional counseling could attenuate muscle mass loss compared with nutritional counseling alone, by adjusting MT for ICW volume in hospitalized patients with unselected cancer.

## 2. Methods

### 2.1. Study Design and Sample Size

This pilot randomized, parallel, and non-blinded trial (registered in the Brazilian Registry of Clinical Trials under code RBR-7z5c63) was conducted with hospitalized patients with unselected cancer, using a convenience sample. The patients were recruited between 2018 and 2019 at Hospital das Clínicas, Goiás, Brazil. This study was approved by the Research Ethics Committee of the Federal University of Goiás at Hospital das Clínicas (approval number 2,916,391). All patients who agreed to participate in the study signed an Informed Consent Form in accordance with Resolution No. 466/12 of the Brazilian Ministry of Health.

### 2.2. Patients and Randomization

The study selection criteria were patients of both sexes, aged ≥ 18 years, with a confirmed diagnosis of any type of cancer, recently admitted to the hospital for hospitalization lasting at least seven days. The average length of stay programmed for the patients was obtained from the medical department. The exclusion criteria were a body mass index (BMI) ≥ 30 kg/m^2^, severe heart disease, in exclusive palliative care, in radiotherapy treatment, failure of follow-up of the intervention protocol, metallic implants (as they can interfere with the conduction of electricity from bioimpedance, and NMES when localized in stimulus region), structured physical activity in the last 6 months, (since it can interfere with muscle sensitivity to neuromuscular stimulation due to the adaptability and quantity of the stimulated muscle), amputees, the presence of epidermal lesion at the stimulation site, and cognitive impairment. Patients who met the inclusion criteria were included to complete the randomization stage. The included patients were randomized in a simple 1:1 allocation ratio, considering the groups: Diet + NMES or to the Diet group.

### 2.3. Experimental Procedure

The study consisted of three steps. In the first step (Day 1), patients were screened for the inclusion criteria, their food intake was evaluated, clinical and socioeconomic data were collected, and muscle thickness (MT) and body composition were assessed using electrical bioimpedance. In the second step (Days 1 to 7), patients in both groups received nutritional counseling, but only one group received the 7-day NMES intervention. The intervention period lasted seven days to reflect the average length of hospital stay among patients with cancer [20]. In the third step (Day 7), food intake, body composition, and MT were reassessed.

### 2.4. NMES and Nutritional Counseling

Both groups received nutritional counseling at the bedside from a trained dietitian during hospitalization. Specific instructions aimed for a minimum intake of 25 kcal/kg of body weight per day of total energy and 1.2 g/kg of body weight per day of protein, achieved through the dietary therapy provided in the hospital environment [9]. However, only the NMES + Diet group received NMES.

The NMES protocol consisted of a 5 min warm-up phase performed at a frequency of 5 Hz with a pulse width of 250 μs [21]. The 30 min muscle stimulation phase was performed at a frequency of 100 Hz with a pulse width of 400 μs [21] (stimulation pattern: 5 s on and 10 s off). NMES was performed twice daily, with a 3 h interval between sessions, for seven consecutive days.

The electrodes were applied to the right and left vastus lateralis and rectus femoris muscles of the quadriceps using a TENS unit (Carci^®^, São Paulo, Brazil; TENSMED IV-4034) [21]. Four 5 × 5 cm self-adhesive electrodes were positioned to ensure even current distribution, allowing both legs to receive stimulation simultaneously. The stimulation amplitude was increased until visible muscle contractions were achieved, according to each patient’s tolerance. Intensity and frequency settings were monitored by trained researchers. Patients who discontinued NMES or missed any session were excluded from the analysis.

### 2.5. Food Intake Evaluation

Food intake was assessed by a dietitian using a 3-day dietary recall during the first step and again during the third step, after the intervention, to evaluate possible changes in food consumption throughout the study. Dietary data were collected in household measures and subsequently converted into gram and milliliter estimates. The dietary information was analyzed using the Dietpro^®^ nutritional analysis software (version 5.8, Viçosa, MG, Brazil). Consumption was evaluated for macronutrients (carbohydrates, lipids, and proteins) and the branched-chain amino acid leucine, using the United States Department of Agriculture (USDA) Food Composition Database [22].

### 2.6. Anthropometric and Body Composition Assessment

BMI (kg/m^2^) was determined based on body weight (kg), obtained using a digital scale (Toledo^®^, Paraná, Brazil) with a precision of 50 g, and height (m), measured using a stadiometer (Sanny^®^, Jacareí, Brazil) with a precision of 0.1 cm.

Body composition was assessed using bioelectrical impedance analysis (BIA) (SECA^®^, model mBCA 525, Hamburg, Germany). This assessment was performed with patients in the supine position (dorsal decubitus) and at rest. The impedance was measured with a current of 100 μA across frequencies ranging from 1 to 1000 kHz. The BIA measurement was taken at eight contact points and evaluated all body segments (right and left sides of the body). The electrodes used for the evaluation were self-adhesive gel electrodes (Kendall™, H59P, Covidien IIc, Mansfield, MA, USA). Each BIA measurement lasted approximately 30 s.

A portable ultrasound device (Bodymetrix^®^ model BX 2000, version 7, Livermore, CA, USA) was used to assess muscle thickness. This assessment was performed by a single trained professional to ensure standardized measurements. The scanning technique was used to evaluate muscle thickness. The muscles assessed were the vastus intermedius and rectus femoris.

Muscle thickness was measured (i) at the junction between the lower and upper two-thirds, and (ii) at the midpoint between the anterior superior iliac spine and the upper pole of the patella. Patients were positioned supine with their legs fully relaxed. The protocol for obtaining thickness values was based on the mean of consecutive measurements taken at the same anatomical site, as recommended by the manufacturer, to improve reliability and minimize random variation, in accordance with Bodymetrix^®^ guidelines.

The assessment was performed with the probe moving at a constant speed, maintaining contact with the skin via ultrasound gel. During the evaluation, muscle thickness images were displayed on a computer using BodyView^®^ software, version 5.3. Each evaluation was performed in triplicate, and the image showing the clearest muscle boundaries was used for analysis. The selected image was divided into eight equal sections, and the mean of these values was recorded as the final muscle thickness.

The MT/ICW ratio was calculated by dividing the value of MT (millimeters) obtained from the ultrasound evaluation by the volume of ICW (liters) obtained from the BIA evaluation.

### 2.7. Socioeconomic, Clinical and Laboratory Data

These data were collected from medical and nutritional records at hospital admission (Day 1) of the intervention period.

### 2.8. Statistical Analyses

The data were entered into Microsoft Excel 2016 spreadsheets for further statistical analysis using MedCalc^®^ software (version 11.1.1.0, Belgium). To identify outlier patients, the Outlier Calculator tool in Prism software (GraphPad Software, ©2024) was used. Normality of data distribution was tested using the *Shapiro–Wilk* test. Variables with a normal distribution were presented as mean ± standard deviation, while non-normally distributed variables were presented as median and interquartile range.

To verify the impact of the intervention on MT and MT/ICW, delta values were calculated by subtracting baseline values from post-intervention measurements. Differences between the Diet and NMES + Diet groups at baseline were analyzed using Student’s *t*-test for normally distributed variables or the Mann–Whitney U test for non-normally distributed variables. Associations between categorical variables were evaluated using the Pearson chi-square test.

ANOVA or *Kruskal–Wallis* tests were performed to assess dietary intake in the Diet and NMES + Diet groups before and after the intervention. To evaluate the outcome variables (MT and MT/ICW), data on sex, age, cancer site, and type of treatment were used as covariates in the ANCOVA test. Adjustments were made for variables that showed significant differences between groups at baseline, as well as for dietary intake variables that differed in the ANOVA before and after the intervention.

For ANCOVA, homogeneity of variances was verified using Levene’s test, and residual distributions were examined for normality. Differences within and between groups were identified through pairwise comparisons using Bonferroni correction as a post hoc test.

The effect size was calculated according to Cohen’s criteria and classified as: <0.2 as “trivial”; >0.2 and <0.5 as “small”; >0.5 and <0.8 as “medium”; and >0.8 as “large”. The significance level adopted for all analyses was *p* < 0.05 [23].

## 3. Results

**Figure 1:**
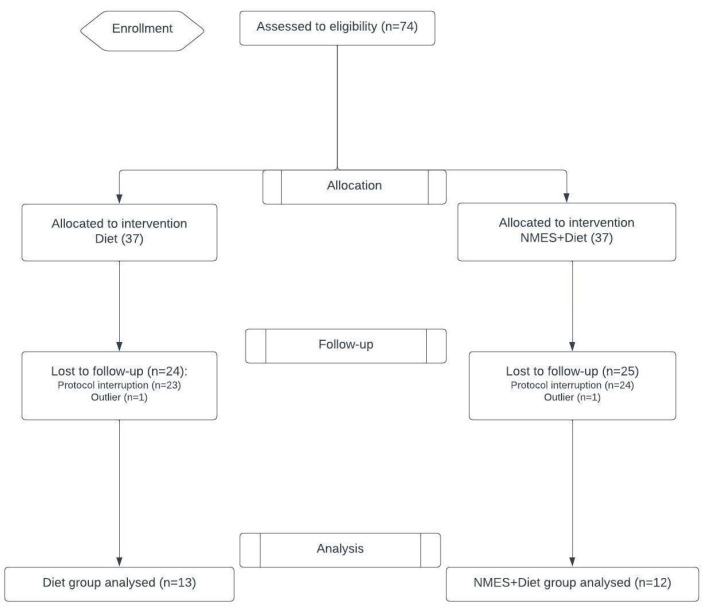
Study flowchart. NMES: neuromuscular electrical stimulation.

A total of 74 patients were eligible for this study. Of these, two were excluded because they were identified as outliers (with muscle thickness gain or loss values far exceeding those of other participants), and 47 did not complete the study due to hospital discharge or withdrawal. Thus, 25 patients (16 with solid tumors and 9 with hematologic tumors) completed the study, with a mean age of 53.8 ± 14.7 years. The participant recruitment flowchart is shown in Figure 1. Both treatment groups (Diet × NMES + Diet) showed similar dropout rates which means participants in the more intensive treatment did not quit more frequently, once intervention with NMES was feasible and well tolerated.

Supplementary Table S1 presents the clinical and oncological treatment types, as well as disease staging. Patients with undefined disease staging had undergone surgical resection, but histopathological and clinical evaluations by specialists had not yet been completed. There were no differences between the groups at baseline, except for the Neutrophil-to-lymphocyte ratio (NLR), NMES + Diet: 0.64 (0.27–3.26) vs. Diet: 4.56 (3.2–19.9), *p* = 0.01 (Table 1).

Table 2 shows significant differences in the assessment of food consumption after ANOVA or *Kruskal–Wallis* analysis. We observed a reduction in carbohydrate intake (*p* = 0.012) and an increase in leucine intake (*p* < 0.001) in both groups post-intervention. However, although leucine intake increased in both groups, the increase was greater in the NMES + Diet group. Similarly, the reduction in carbohydrate intake was also more pronounced in the NMES + Diet group (Table 2).

**Figure 2:**
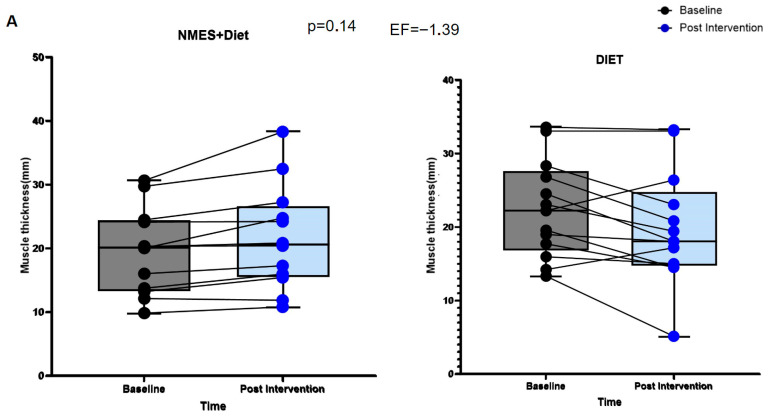

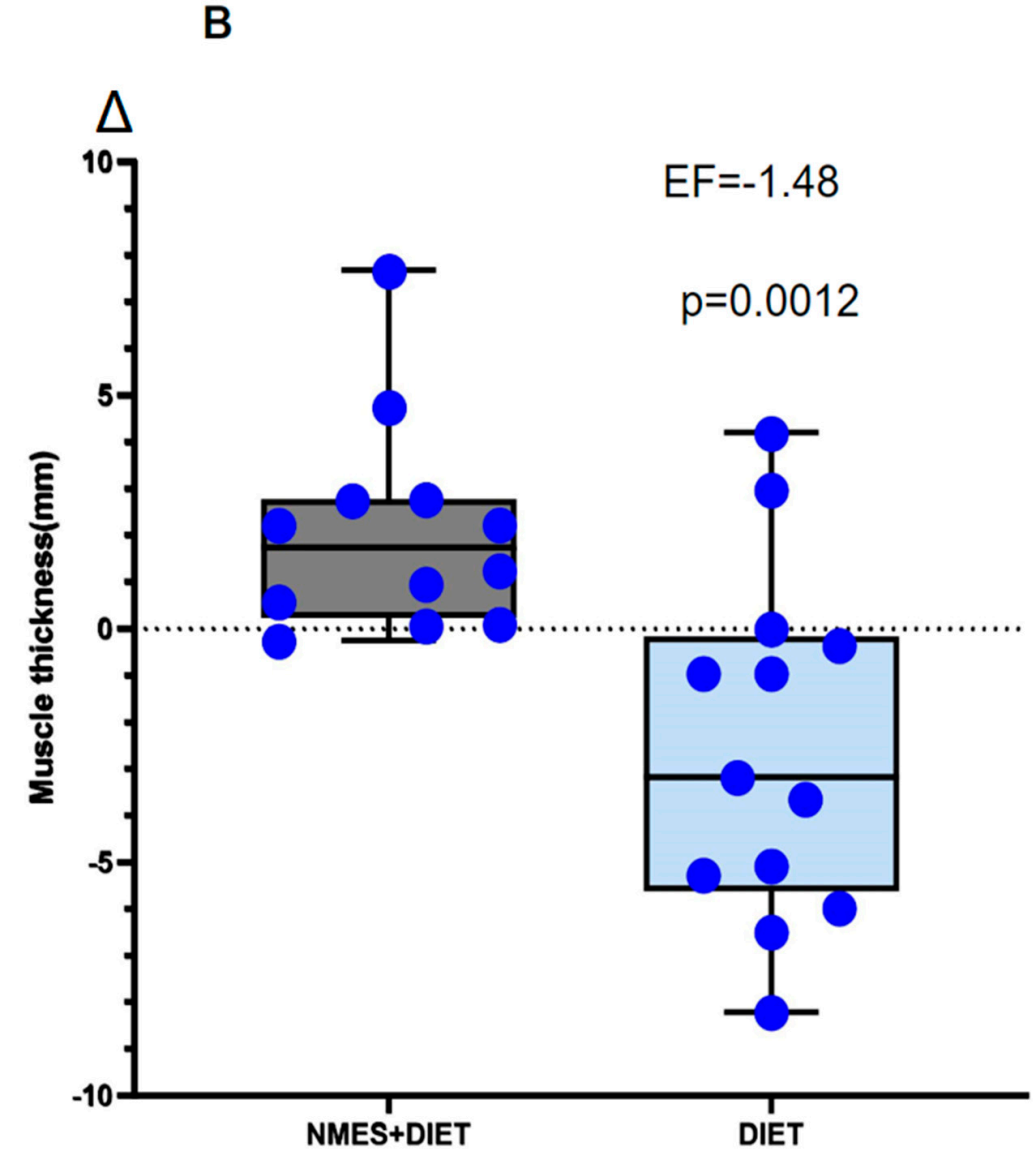
(**A**): Muscle thickness (mm) pre and post intervention (Adjustment by age, sex, type of treatment, type of cancer, baseline NLR values, consumption of carbohydrates (%) and leucine (g). (**B**): Delta of muscle thickness (mm) for both groups. Diet group: ∆: −2.53 ± 3.73 mm vs. NMES + Diet group: ∆: 2.09 ± 2.27 mm, *p* = 0.001. EF: effect size (D-cohen: 1.48; CI95% 0.62–2.26).

Figure 2A shows that there were no significant differences between the groups in pre- and post-intervention MT values (*p* = 0.14). However, as shown in Figure 2B, the delta analysis revealed that the NMES + Diet group exhibited an increase in MT, whereas the group receiving only nutritional counseling showed a decrease in muscle thickness.

**Figure 3:**
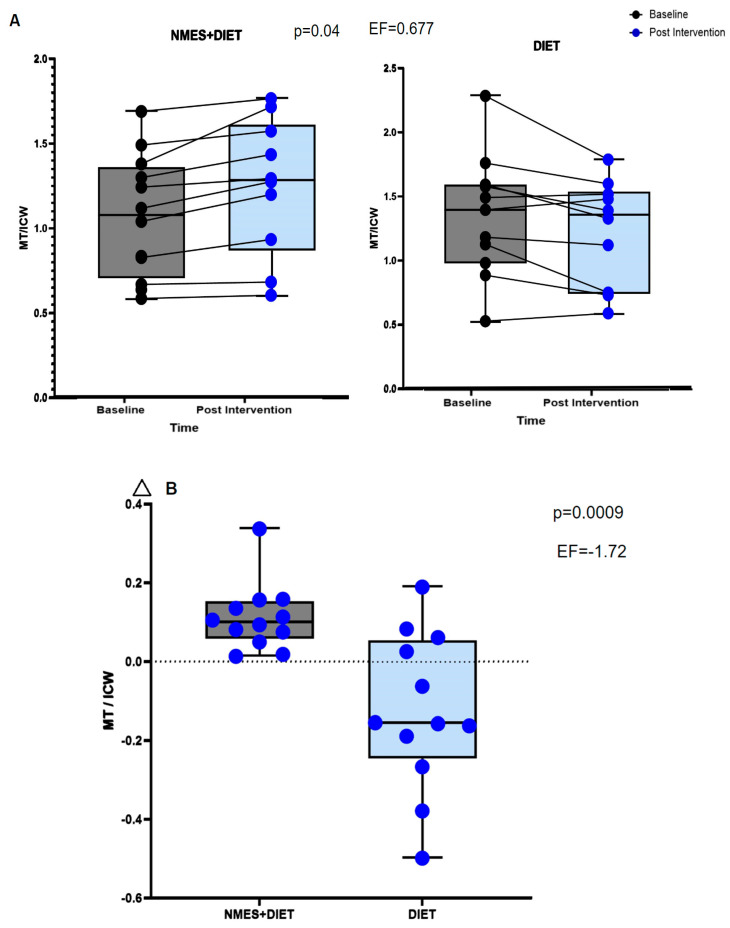
(**A**): Muscle thickness (MT)/intracellular water (ICW) ratio (mm/L) pre and post intervention (Adjustment by age, sex, type of treatment, type of cancer, baseline NLR values, consumption of carbohydrates (%) and leucine (g). MT/ICW, were lower in the NMES + Diet group at baseline (*p* = 0.04). (**B**): Delta of MT/ICW ratio (mm/L) for both groups. EF: effect size.

Figure 3A shows that, at baseline, MT/ICW values were lower in the NMES + Diet group (*p* = 0.04), but there were no significant changes in MT/ICW post-intervention in either group (*p* > 0.05). However, as shown in Figure 3B, the delta analysis indicated that the NMES + Diet group had higher MT/ICW values, reflecting an increase after the intervention, whereas the Diet group showed a reduction (Diet group: ∆ = −0.15 ± 0.19 mm/L vs. NMES + Diet group: ∆ = 0.11 ± 0.09 mm/L, *p* < 0.001).

## 4. Discussion

Our findings suggest that this intervention promoted a greater increase in muscle thickness compared with nutritional counseling alone. Furthermore, when we evaluated the delta of the MT/ICW ratio, we found that the NMES + Diet group showed a higher MT/ICW ratio post-intervention than the Diet group. Our hypothesis is that the NMES + Diet intervention promotes an increase in muscle thickness, but to a smaller extent than the corresponding increase in ICW. This differs from the Diet group, in which the reduction in MT occurred in a greater proportion than the decrease in ICW, as the delta of the MT/ICW ratio showed a post-intervention decline. This is the first study to evaluate both muscle thickness and the MT/ICW ratio in an intervention combining NMES with nutritional counseling.

The increase in the MT/ICW ratio after the intervention in the NMES + Diet group may be explained by the fact that the increase in muscle thickness occurred in a greater proportion than the change in ICW, likely due to an increase in extracellular water (ECW) within the muscle resulting from cell damage induced by NMES. As suggested by the study by Taniguchi et al. (2020) [24], performing multiple sets of exhaustive knee extensions led to a significant increase in both the extracellular water-to-intracellular water ratio and muscle thickness in these individuals [24]. The intracellular fluid volume of muscle fibers is tightly regulated by complex homeostatic mechanisms. Thus, the intracellular fluid volume typically returns to baseline shortly after exercise [25], indicating that longer-term interventions are required to detect sustained changes in these parameters [24].

The reduction in the MT/ICW ratio in the Diet group may have resulted from a greater decrease in MT than in ICW. Physical inactivity associated with severe illness are key factors that influence the inflammatory and metabolic stress states, leading to hormonal alterations such as low insulin and elevated glucagon and catecholamine levels, which may independently contribute to cellular atrophy and a reduction in cell mass [26,27,28]. In our study, it is likely that ICW volume remained constant during the intervention period, even with the observed reduction in muscle thickness. This finding corroborates previous literature indicating that intracellular volume initially remains stable, even under conditions of critical illness, and that reductions in intracellular volume occur progressively over time [5,29].

Data from the literature indicate that the application of NMES, when combined with adequate nutritional intake, can increase both postabsorptive and postprandial muscle protein synthesis rates, thereby preventing muscle loss and promoting lean mass gain [8,30]. Among the potential mechanisms underlying the NMES-induced increase in muscle protein synthesis, enhancement of myocellular mTOR signaling and reversal of the skeletal muscle mRNA expression of ligases involved in the ubiquitin–proteasome pathway have been proposed [30]. While previous studies have reported positive effects of NMES on muscle mass and strength, no studies to date have examined NMES in conjunction with controlled dietary intake in such a vulnerable patient population [14,15,16,17,18].

In our study, patients in both groups were encouraged to increase their overall dietary intake, particularly protein intake, in accordance with the ESPEN guidelines [9]. We observed an increase in leucine intake post-intervention in both groups. However, leucine intake was higher in the NMES + Diet group after the intervention compared with the Diet group. In addition, carbohydrate intake (%) decreased in both groups, with a greater reduction observed in the NMES + Diet group. These differences may have clearly contributed to the relatively greater loss of thigh muscle thickness in the Diet group and to the effectiveness of the NMES + Diet intervention. Indeed, consuming a high-protein diet is essential for stimulating muscle protein synthesis [31]. Although dietary protein supports muscle hypertrophy, protein intake alone—without muscle contraction—appears insufficient to adequately stimulate muscle protein synthesis and maintain muscle mass [7]. Kilroe et al. (2021) [32] demonstrated that protein intake alone does not significantly modulate muscle protein synthesis rates during periods of immobilization in healthy individuals. Hence, in addition to consuming an adequate amount of food, especially protein, exercise or exercise surrogates are required to effectively stimulate muscle protein synthesis [33]. Muscle mass is regulated by protein balance and physical activity, where exercise performed prior to protein intake enhances the food-induced stimulation of protein synthesis, resulting in a positive protein balance [34].

Studies have shown that repetitive muscle contractions can induce muscle damage, leading to myofiber necrosis, ultrastructural disruption, edema, and inflammation [35,36,37,38]. Muscle inflammation typically peaks three days after exercise, followed by the onset of muscle fiber regeneration approximately seven days later [35,39,40].

Schink et al. (2018) [41] performed whole-body electromyostimulation for 12 weeks combined with individualized nutritional support in patients with advanced cancer and found an increase in skeletal muscle mass in the intervention group compared with the dietary intervention alone. Our study was a short-term intervention, and NMES was applied only to the lower limbs. We observed that a period of disuse—likely accompanied by additional catabolic factors—during a typical 7-day hospital stay resulted in a substantial loss of muscle thickness. Furthermore, in our study, the NMES + Diet group received the intervention for seven days; however, although we controlled dietary intake, we cannot conclude that the increase in muscle thickness observed in our patients was exclusively due to hypertrophy, given the short duration of the intervention. The increase in delta MT may also have been influenced by muscle damage induced by NMES, which can lead to an increase in extracellular water [34]. Since the post-intervention muscle assessment in our study was performed shortly after the intervention period, it is possible that the muscle was still in the inflammatory phase preceding regeneration.

The positive aspects of our study include: (i) the control of dietary intake in hospitalized patients; (ii) adjustment for factors that could influence changes in muscle mass before and after the intervention, such as age, sex, type of treatment, type of cancer, inflammation (assessed by NLR), and food intake; (iii) in addition to assessing muscle thickness by ultrasound to determine gains or losses in muscle mass during the intervention, we also evaluated the relationship between muscle thickness and intracellular water, which provides a more accurate reflection of muscle quality and the hypertrophy process.

The study has several limitations that require acknowledgment: (i) the small sample size, as this was a pilot study; (ii) the heterogeneity of cancer types (solid and hematologic); (iii) the study was conducted in hospitalized cancer patients; therefore, in addition to disuse itself, muscle loss may also have been influenced by the disease’s pathophysiology; (iv) dietary intake was assessed using three 24 h dietary recalls, which may not accurately reflect true intake and could, for example, lead to an overestimation of leucine consumption; (v) the study was not double-blinded, as participants were exposed to electrical stimuli with progressive intensity according to their tolerance, which was controlled by the operator; (vi) the constancy of ICW was assumed rather than directly measured, representing a notable methodological limitation. Although this assumption may be plausible in certain contexts, it remains speculative and could compromise the robustness of physiological inferences drawn from this parameter. Therefore, future studies are encouraged to perform direct and accurate ICW assessments using validated methods such as isotope dilution—particularly in clinical populations or conditions in which variations in cellular water content could significantly influence the analyzed outcomes. However, we do not consider this to be a major limitation, as this approach allows for appropriate progression of the stimulation intensity.

In conclusion, we found that the short-term (7-day) intervention involving nutritional counseling plus NMES led to an increase in thigh muscle thickness and in the ratio of muscle thickness to intracellular water volume, which may have been influenced by an increase in ECW following NMES. Additional studies with controlled dietary intake are needed to investigate whether longer-term interventions can promote increases in ICW proportional to those in MT, thereby improving muscle quality in hospitalized cancer patients. The short duration of this pilot study limits the generalizability of the findings, highlighting the need for further research in this area.

In addition, our findings demonstrate that stimulating muscle groups combined with dietary and nutritional counseling can positively influence body composition, promoting gains in muscle thickness and intracellular water even during short hospital stays. Therefore, such practices could be incorporated into routine hospital care and implemented by trained professionals to improve patient prognosis.

## Figures and Tables

**Table 1:** Baseline characteristics of the participants.

Variables	Diet + NMES (n = 12)	Diet (n = 13)	*p* Value
Age (years)	54.0 ± 16.1	53.5 ± 14.0	0.92
Sex (n,%)			
Female	5 (41.7)	7 (53.8)	0.83
Male	7 (58.3)	6 (46.2)	
Performance status (n,%)			
1	2 (16.7)	0 (0.0)	0.24
2	4 (33.3)	7 (53.8)	
3	6 (50.0)	6 (46.2)	
4	0 (0.0)	0 (0.0)	
Cancer location (n, %)			
Gastrointestinal	4 (33.3)	10 (76.9)	0.07
Hematological	7 (58.3)	2 (15.4)	
Reproductive system	1 (8.3)	1 (7.7)	
Cancer treatment (n, %)			
Chemotherapy	3 (25.0)	2 (15.4)	0.17
Pre-surgical	3 (25.0)	8 (61.5)	
Clinical	6 (50.0)	3 (23.1)	
Functional mobility (n,%)			
Limited mobility	6 (50.0)	4 (30.8)	0.56
No limited mobility	6 (50.0)	9 (69.2)	
Phase Angle (°)	4.7 ± 1.08	4.2 ±0.71	0.15
ECW/TBW (%)	46.71 ± 3.66	48.54 ± 2.72	0.19
MT (mm)	19.5 ± 6.8	22.4 ± 6.6	0.29
MT/ICW (mm/L)	1.06 ± 0.36	1.34 ± 0.47	0.13
Lean Body Mass (kg)	47.0 ± 11.0	47.2 ± 12.0	0.97
Biochemical data			
Hemoglobin (g/dL)	9.1 ± 2.5	10.3 ± 2.7	0.30
Creatinine (mg/dL)	0.8 (0.8–0.9)	0.9 (0.7–1.5)	0.75
Albumin (g/dL)	3.2 ± 0.7	3.4 ± 0.9	0.52
NLR	0.64 (0.3–3.3)	4.56 (3.2–19.9)	0.01 *
CRP (mg/dL)	1.1 (0.3–8.0)	4.7 (0.4–18.8)	0.12

NLR: Neutrophil-to-lymphocyte ratio. CRP: C-reactive protein (mg/dL). ECW/TBW: ratio of Extracellular Water to Total Body Water (%). MT: muscle thickness (mm). MT/ICW: ratio of muscle thickness relative to intracellular water volume (mm/L). * *p* < 0.05.

**Table 2:** Assessment of food consumption pre and post intervention.

Variables	Diet + NMES Group		Diet Group		*p* Value	EF ^a^
	Baseline	Post-Intervention	Baseline	Post-Intervention		d-Cohen
Calories (kcal/kg b.w./day)	21.2 ± 7.3	27.8 ± 4.6	21.5 ±13.1	23.3 ± 9.0	0.27	0.37
Carbohydrate (%)	59.8 (51.0–66.1)	48.0 (48.0–52.2) ^§^	51.6 (48.0 −59.5)	46.8 (45.3–52.0) ^§ β^	0.012	−0.23
Protein (%)	16.4 (13.0–18.5)	15.5 (15.5–19.6)	17.9 (15.3–20.0)	20.0(16.4.−21.5)	0.17	−0.04
Protein (g/kg b.w./day)	0.86 ± 0.38	1.1 ± 0.24	1.09 ± 0.89	1.0 ± 0.6	0.58	1.42
Leucine (g)	3.0 ± 1.3	5.9 ± 1.9 ^§ β^	2.1 ± 1.6	4.4 ± 1.9 ^§ β^	<0.001	0.54
Lipids (%)	23.4 (20.4–30.2)	23.2 (23.2–28.1)	31.2 (23.9–34.8)	31.9 (23.0–34.8)	0.16	0.08

^a^ EF: effect size. dKorr sensu Klauer (2001). Difference intra group: NMES + Diet and Diet before and after the intervention = ^§^. Difference between groups NMES + Diet and Diet group after the intervention = ^β^.

## Data Availability

The datasets generated and/or analyzed during the current study are available from the corresponding author on reasonable request.

## Supplementary Materials

The following supporting information can be downloaded at https://www.mdpi.com/article/10.3390/pathophysiology32040068/s1: Supplementary Table S1. Characterization of the type of treatment and staging per patient.
