# Supplementary material for: Neuromuscular Electrical Stimulation Plus Nutritional Counseling Attenuates Thigh Muscle Thickness Loss in Hospitalized Cancer Patients
Source: Pathophysiology. 2025 Dec 2;32(4):68. doi: 10.3390/pathophysiology32040068 (PMC12735726; doi:10.3390/pathophysiology32040068)
Supplement: Supplementary file 1 [file pathophysiology-32-00068-s001.zip › pathophysiology-3639232-supplementary.pdf]

Supplementary Table S1. Characterization of the type of treatment and staging per patient.

**Patients NMES + Diet**	**Oncological treatment**	**Cancer staging**
1	Clinical treatment of symptoms	Undefined
2	Alkylating chemotherapy	Undefined
3	Clinical treatment of symptoms	Undefined
4	Alkylating chemotherapy	Undefined
5	Clinical treatment of symptoms	Undefined
6	Pre-surgery: biliodigestive bypass	IV
7	Chemotherapy with endocrine therapy	II
8	Clinical treatment of symptoms	Undefined
9	Clinical treatment of symptoms	Undefined
10	Pre surgical: Total gastrectomy + Y Roux	II
11	Pre-surgical: subtotal gastrectomy + Y Roux	III
12	Clinical treatment of symptoms	IV
**Patients Diet group**		
1	Chemotherapy with antimetabolites	Undefined
2	Clinical treatment of symptoms	Undefined
3	Pre surgery: Biliodigestive bypass	IV
4	Pre-surgery: proctectomy	Undefined
5	Pre surgical: biopsy	Undefined
6	Clinical treatment of symptoms	Undefined
7	Pre surgical: Total gastrectomy + Y Roux	Undefined
8	Pre surgical: pancreatectomy	Undefined
9	Pre surgical: Right colectomy with ileotransverse anastomosis	Undefined
10	Pre surgical: right colectomy	Undefined
11	Alkylating chemotherapy	Undefined
12	Pre surgical: biopsy	Undefined
13	Clinical treatment of symptoms	Undefined
